# Vaccenic and Elaidic Acid Modify Plasma and Splenocyte Membrane Phospholipids and Mitogen-Stimulated Cytokine Production in Obese Insulin Resistant JCR: LA-*cp* Rats 

**DOI:** 10.3390/nu2020181

**Published:** 2010-02-11

**Authors:** Megan R. Ruth, Ye Wang, Howe-Ming Yu, Susan Goruk, Martin J. Reaney, Spencer D. Proctor, Donna F. Vine, Catherine J. Field

**Affiliations:** 1 Boston University School of Medicine, 72 East Concord Street, Robinson 4400, Boston, MA, 02118, USA; Email: mruth@bu.edu; 2 Alberta Institute for Human Nutrition, University of Alberta, Edmonton, AB, T6G 2P5, Canada; Email: flora.wang@ualberta.ca (Y.W.), howeming@ualberta.ca (H.-M.Y), sue.goruk@ualberta.ca (S.G.), spencer.proctor@ualberta.ca (S.D.P.), donna.vine@ualberta.ca (D.F.V.),; 3 Department of Food and Bioproduct Sciences, University of Saskatchewan, Saskatoon, SK, S7N 5A8, Canada; Email: martin.reaney@usask.ca

**Keywords:** vaccenic acid, elaidic acid, phospholipid, obese, immune, inflammation, cytokines, *trans* fat

## Abstract

This study assessed the long-term effects of dietary vaccenic acid (VA) and elaidic acid (EA) on plasma and splenocyte phospholipid (PL) composition and related changes in inflammation and splenocyte phenotypes and cytokine responses in obese/insulin resistant JCR:LA-*cp* rats. Relative to lean control (Ctl), obese Ctl rats had higher serum haptoglobin and impaired T-cell-stimulated cytokine responses. VA and EA diets improved T-cell-stimulated cytokine production; but, only VA normalized serum haptoglobin. However, EA- and VA-fed rats had enhanced LPS-stimulated cytokine responses. The changes elicited by VA were likely due changes in essential fatty acid composition in PL; whereas EA-induced changes may due to direct incorporation into membrane PL.

## 1. Introduction

It is well established that the type and total amount of dietary fat can influence immunity and disease risk throughout the lifespan [1]. Consumption of industrial produced *trans* fatty acids (TFA), and more specifically the major TFA in hydrogenated fats, elaidic acid (EA, 18:1*trans*9), is associated with an increased risk of cardiovascular disease (CVD) [2,3,4]. It appears that TFA may negatively impact blood lipid profiles and endothelial function [5]. More recently, a positive association between dietary TFA and systemic inflammation was reported in healthy women [6,7], suggesting a negative effect of TFA on immune function. Consistently, a short-term (5 wk) clinical trial in healthy men, reported that consuming a diet containing 8% of energy as a mixture of TFA (unspecified composition) resulted in an increase in systemic concentrations of C-reactive protein [8]. Additionally, Han *et al.* (2002) reported that peripheral blood mononuclear cells of overweight subjects fed partially-hydrogenated margarine, but not those fed butter fat, produced more IL-6 and TNF-α in response to lipopolysaccharide (LPS) [9]. Together these studies suggest that partially hydrogenated fat, which contains a mixture of 18-carbon TFA, enhances the inflammatory response. However, there is little known about the health implications of individual and natural TFA. 

Obesity is one of the leading health crises facing the global community, particularly due to the increased risk of inflammatory-associated disease, including type 2 diabetes [10], CVD [11] and cancer [12,13]. Altered immune responses and elevations in inflammatory mediators have been reported in obese individuals and are implicated in the pathogenesis of these co-morbidities. Alterations in the innate and acquired immune systems have also been reported in obese humans and rodents. In overweight adults greater LPS-induced TNF-α production [14], higher NF-κB binding activity and inflammatory cytokine expression [15], and reduced proliferative responses of B and T-lymphocytes [16,17,14] have been observed. Furthermore, we have reported that immune cells from genetic rodent models of obesity have reduced T-cell responses [18] and greater inflammatory cytokine production [18,19]. 

The JCR:LA-*cp* rat has a defect in the Ob gene rendering the model to have a dysfunctional leptin receptor leading to hyperphagia and obesity. Rats that are homozygous for the autosomal recessive *cp* gene (*cp*/*cp*) are obese and insulin resistant, have dyslipidemia and develop atherosclerosis and have been used extensively to study the pathogenic mechanisms and risk factors of CVD and pre-diabetes [21,22,23,24]. We have also determined that this model has altered inflammatory and immune responses and that modifying dietary fatty acid composition can alter these abnormalities [19,25,26]. We have demonstrated that diets enriched in long chain (n-3) polyunsaturated fatty acids (PUFA) can improve mitogen stimulated cytokine production (IL-1β, IL-4 and IL-10) in JCR:LA-*cp* rodents at least in part through modification of cellular membrane fatty acid composition [19,26]. This exemplifies that the pre-disposition to immune dysfunction in obesity can be altered by modifying dietary fatty acid composition.

Current labeling legislation in both Canada and the United States group the naturally occurring fatty acid, vaccenic acid (VA, 18:1*trans*11), which is also produced during hydrogenation, with all other TFA. Our recent work [27,28] and that of others [29], demonstrated that VA can lower fasting triglycerides, total cholesterol, low density lipoprotein and non-esterified fatty acids in animal models of dyslipidemia. We have also reported that short-term (3 wk) supplementation of VA (1.5% w/w) normalized stimulated IL-2 and TNF-α production and increased IL-10 production in the JCR-LA-*cp* rat [25], which may be due to incorporation of VA into immune cell phospholipids (PL). Modification of immune cell PL fatty acid composition induces structural and functional changes that can alter inflammatory and T-cell responses [30]. 

Despite current regulations that group all industrial produced TFA with VA, evidence suggests a difference between the biological activities of individual TFA. Although TFA are a constituent of most North American diets, long-term feeding studies have not been conducted to determine the effects of individual TFA on inflammatory and immune processes in the obese, insulin resistant state. Therefore, the objectives of this study were to compare the effects of long-term feeding of the two major dietary TFA, VA and EA, on immune function in insulin-resistant obese JCR-LA-*cp* rodent model and to determine if their biological effects are mediated through incorporation into plasma and membrane PL.

## 2. Results and Discussion

### 2.1. Body weight and Spleen Characteristics

In all dietary groups, obese rats were shown to have higher body (650 ± 9 g vs. 382 ± 7 g, P < 0.001) and spleen weights compared (0.77 ± 0.01g, P < 0.05) to lean Ctl rats. Dietary treatment did not significantly affect final body weight (650 ± 9 g, Ctl; 660 ± 11 g, VA; 668 ± 14 g, EA) or percent body weight gain (48.9%, Ctl; 49.6%, VA; 52.7%, EA) among obese rats. Spleens of rats fed EA (1.2 ± 0.02 g) weighed significantly more than obese Ctl rats (1.0 ± 0.04 g); while the spleens of VA-fed rats (0.88 ± 0.03 g) weighed significantly less compared to obese Ctl and EA-fed rats (P < 0.05). The number of splenocytes did not differ between lean (3.52 × 10^8^ cells) and obese (3.90 × 10^8^ cells) or with VA (3.97 × 10^8^ cells) or EA (3.00 × 10^8^ cells) feeding. 

### 2.2. Fatty Acid Composition of Plasma PL

Compared to lean Ctl rats, obese Ctl rats had a higher proportion of 16:0, 18:1 (n-9), C18:1 (n-7), 20:3 (n-6), total MUFA, and a lower percentage of 18:0 and 20:4 (n-6) in plasma phospholipids (Table 1, P < 0.05). Compared to obese Ctl and EA rats, there was a higher proportion of 18:1*trans*11, 18:1 (n-9), C18:1 (n-7), 18:2 (n-6) and 20:3(n-6), and a lower percentage of 12:0 (Ctl only) in plasma PLs of rats fed VA (Table 1, P < 0.05). Overall VA-fed rats had a higher percentage of total PUFA, n-6:n-3 PUFA, n-6 PUFA and PUFA:SFA, and a lower proportion of total SFA relative to EA-fed rats (Table 1, P < 0.05). EA-fed rats also had a higher proportion of 18:1 *trans*9 compared to all groups, and a lower proportion of total n-6 PUFA relative to obese Ctl rats (Table 1, P < 0.05). The plasma level of EA in EA-fed rats was similar to the plasma level of VA in VA-fed rats. The plasma 20:4(n-6):20:3(n-6), an estimate of delta-5 desaturase, was higher in VA-fed rats (0.14 ± 0.00) relative to EA (0.11± 0.00) and Ob Ctl rats (0.12 ± 0.00). There was a slight difference in estimated delta-9 desaturase activity (MUFA:SFA) between VA (4.48 ± 0.20) and Ctl (6.85 ± 0.58), but not EA (5.39 ± 0.85). 

**Table 1:** Plasma PL fatty acid concentrations in lean rats and obese JCR:LA-*cp* rats fed Ctl, VA or EA diets.

	Lean Ctl	Obese Ctl	VA	EA
	% of fatty acids			
**12:0**	2.3 ± 0.2^ab^	2.7 ± 0.5^a^	1.0 ± 0.3^b^	1.8 ± 0.9^ab^
**16:0**	21 ± 1^b^	26 ± 1^a^	25 ± 1^a^	28 ± 3.1^a^
**18:0**	34 ± 1^a^	27 ± 0^b^	26 ± 1^b^	28 ± 3^b^
**18:1*trans*9**	0^b^	0^b^	0^b^	1.6 ± 0.2^a^
**18:1*trans*11**	0^b^	0^b^	1.5 ± 0.1^a^	0^b^
**18:1(n-9)**	3.9 ± 0.1^c^	5.4 ± 0.1^b^	5.9 ± 0.1^a^	5.2 ± 0.1^b^
**18:1(n-7)**	0.7 ± 0.0^c^	1.4 ± 0.0^b^	1.6 ± 0.1^a^	1.3 ± 0.1^b^
**18:2(n-6)**	15 ± 1^b^	18 ± 0.9^b^	22 ± 1^a^	15 ± 2^b^
**c9t11CLA**	0	0	0.06 ± 0.05	0
**20:3(n-6)**	0^c^	1.8 ± 0.3^b^	2.4 ± 0.1^a^	2.0 ± 0.3^ab^
**20:4(n-6)**	18 ± 1^a^	14 ± 1^b^	11 ± 1^b^	10 ± 1^b^
**22:5(n-3)**	0.05 ± 0.05^a^	0.24 ± 0.01^a^	0.31 ± 0.05^a^	0.18 ± 0.06^a^
**22:6(n-3)**	1.3 ± 0.1^a^	1.5 ± 0.1^a^	1.1 ± 0.1^a^	1.3 ± 0.2^a^
**MUFA**	4.3 ± 0.2^c^	6.7 ± 0.1^ab^	7.5 ± 0.1^a^	6.4 ± 0.1^b^
**SFA**	56 ± 1^a^	55 ± 1^ab^	52 ± 0^b^	57 ± 1^a^
**PUFA**	34 ± 2^ab^	35 ± 1^ab^	36 ± 0^a^	32 ± 1^b^
**PUFA:SFA**	0.61 ± 0.20^ab^	0.64 ± 0.03^ab^	0.69 ± 0.01^a^	0.56 ± 0.02^b^
**n-6 PUFA**	33 ± 1^a^	33 ± 1^a^	35 ± 0^a^	30 ± 1^b^
**n-3 PUFA**	1.4 ± 0.1^a^	1.7 ± 0.1^a^	1.4 ± 0.1^a^	1.7 ± 0.2^a^
**n-6:n-3 PUFA**	26 ± 2.7^a^	20 ± 1.1^ab^	26 ± 2.9^a^	19 ± 2.5^b^

Data represent mean ± SEM (n=8/group). Means within the same row that do not share a common letter are significantly different (P < 0.05). The relative proportions of fatty acids from 12:0 to 24:1(n-9) were measured, but only the major fatty acids were reported. Abbreviations used: ND, not detectable; SFA, sum of saturated fatty acids; MUFA, sum of monounsaturated fatty acids; (n-6) PUFA, sum of (n-6) polyunsaturated fatty acids; (n-3) PUFA, sum of (n-3) polyunsaturated fatty acids.

### 2.3. Fatty Acid Composition of Splenocyte PL

In the PE fraction, splenocytes of obese Ctl rats had a higher proportion of 16:0, 18:1*cis*11, 20:3(n-6), 22:5(n-3), 22:6(n-3), MUFA and n-3 PUFA and had a lower percentage of 18:0, 18:2(n-6), (n-6) PUFA and (n-6):(n-3) compared to lean rats (Table 2, P < 0.05). Relative to obese Ctl rats, rats fed VA had a higher proportion of VA, 18:1*cis*11, and 18:2(n-6) and a lower percentage of 20:4(n-6), 22:6(n-3) and total PUFA (P < 0.05, Table 2). Rats fed EA had a higher percentage of EA and 20:3(n-6) and a lower proportion of 16:0, 18:1*cis*11 and total SFA (P < 0.05, Table 2).

**Table 2:** Splenocyte phosphatidylethanolamine fatty acid composition of lean rats and obese JCR:LA-*cp* rats fed Ctl, VA or EA diets.

	Lean Ctl	Obese Ctl	VA	EA
	g/100g lipid			
**C16:0**	3.4 ± 0.1^c^	5.0 ± 0.1^a^	5.2 ± 0.2^a^	4.2 ± 0.1^b^
**C18:0**	25 ± 0^a^	22 ± 0^b^	22 ± 0^b^	23 ± 0^b^
**C18:1*trans*9**	0	0	0	1.4 ± 0.1
**18:1*trans*11**	0.38 ± 0.08^b^	0.34 ± 0.02^b^	1.1 ± 0.1^a^	0.30 ± 0.00^b^
**C18:1*cis*9**	5.0 ± 0.1^a^	5.2 ± 0.1^a^	5.2 ± 0.1^a^	5.1 ± 0.1^a^
**C18:1*cis*11**	0.58 ± 0.03^d^	1.0 ± 0.0^b^	1.2 ± 0.0^a^	0.91 ± 0.03^c^
**C18:2 (n-6)**	4.3 ± 0.4^a^	3.6 ± 0.1^b^	4.1 ± 0.1^a^	3.5 ± 0.1^b^
**C18:3 (n-3)**	0.30 ± 0.02^a^	0.29 ± 0.03^a^	0.35 ± 0.01^a^	0.31 ± 0.01^a^
**C20:3 (n-6)**	1.0 ± 0.1^c^	1.5 ± 0.1^b^	1.5 ± 0.0^b^	1.7 ± 0.1^a^
**C20:4 (n-6)**	40 ± 1^a^	40 ± 0^a^	38 ± 1^b^	40 ± 0^a^
**C20:5 (n-3)**	0.76 ± 0.46^a^	0.40 ± 0.12^a^	1.4 ± 0.7^a^	0.68 ± 0.15^a^
**C22:4 (n-6)**	0.65 ± 0.06^a^	0.70 ± 0.05^a^	0.62 ± 0.07^a^	0.55 ± 0.03^a^
**C22:5 (n-3)**	2.7 ± 0.1^b^	4.4 ± 0.1^a^	4.1 ± 0.1^a^	4.3 ± 0.2^a^
**C22:6 (n-3)**	2.1 ± 0.1^c^	3.9 ± 0.2^a^	2.8 ± 0.1^b^	3.6 ± 0.1^a^
**MUFA**	5.7 ± 0.3^b^	6.5 ± 0.1^a^	7.5 ± 0.1^a^	6.4 ± 0.1^a^
**SFA**	33 ± 1^a^	31± 0^a^	31 ± 1^a^	29 ± 0^b^
**PUFA**	60 ± 1^ab^	61 ± 0^a^	58 ± 1^b^	61 ± 0^a^
**PUFA:SFA**	1.8 ± 0.1^b^	2.0 ± 0.0^ab^	1.9 ± 0.1^b^	2.1 ± 0.0^a^
**(n-6) PUFA**	46 ± 1^a^	44 ± 0.2^bc^	43 ± 1^c^	45 ± 0^ab^
**(n-3) PUFA**	14 ± 0^c^	16 ± 0^a^	15 ± 1^b^	16 ± 0^ab^
**(n-6):(n-3) PUFA**	3.3 ± 0.1^a^	2.7 ± 0.0^b^	2.9 ± 0.1^b^	2.8 ± 0.1^b^

Data represent mean ± SEM (n = 8/group). Means within the same row that do not share a common letter are significantly different (p < 0.05). The relative proportions of fatty acids from 12:0 to 24:1(n-9) were measured, but only the major fatty acids were reported. Abbreviations used: SFA, sum of saturated fatty acids; MUFA, sum of monounsaturated fatty acids; (n-6) PUFA, sum of (n-6) polyunsaturated fatty acids; (n-3) PUFA, sum of (n-3) polyunsaturated fatty acids.

In the PC fraction, splenocytes of obese Ctl rats had a higher proportion of 16:0, 18:1*cis*9*, *18:1*cis*11, 20:3(n-6), 22:6(n-3) MUFA and n-3 PUFA and a lower percentage of 18:0, 18:2(n-6), 18:3(n-3), 20:5(n-3), C22:4(n-6), total PUFA, (n-6) PUFA and (n-6):(n-3) compared to lean Ctl rats (Table 3, P < 0.05). Relative to obese Ctl rats, both VA and EA fed rats had a higher proportion 18:2(n-6) and a lower proportion of 20:4(n-6) (Table 3, P < 0.05). Only rats fed VA had a higher proportion of VA, and (n-6):(n-3) PUFA and lower proportion of 18:1*cis*9, 22:6(n-3) and (n-3) PUFA relative to obese Ctl group (P < 0.05, Table 3). The EA group had more EA, 18:2(n-6), 20:3(n-6), and 22:5(n-3) and less 18:0, VA, and total SFA in PC PL compared to obese Ctl rats (P < 0.05, Table 3).

**Table 3:** Splenocyte phosphatidylcholine fatty acid composition of lean rats and obese JCR:LA-*cp* rats fed Ctl, VA or EA diets.

	Lean Ctl	Obese Ctl	VA	EA
	g/100g lipid			
**C16:0**	31 ± 0^a^	36 ± 0^b^	37± 0^b^	36 ± 0^b^
**C18:0**	17 ± 0^a^	13 ± 0^b^	12 ± 0^bc^	12 ± 0^c^
**C18:1*trans*9**	0	0	0	2.1 ± 0.0
**18:1*trans*11**	0.60 ± 0.07^b^	0.71 ± 0.04^b^	1.5 ± 0.1^a^	0
**C18:1*cis*9**	9.0 ± 0.1^b^	9.4 ± 0.1^a^	9.0 ± 0.1^b^	9.6 ± 0.1^a^
**C18:1*cis*11**	2.0 ± 0.1^c^	3.4 ± 0.1^ab^	3.6 ± 0.1^a^	3.4 ± 0.0^b^
**C18:2 (n-6)**	12 ± 0^a^	10 ± 0^c^	11 ± 0^b^	11 ± 0^b^
**C18:3 (n-3)**	0.95 ± 0.02^a^	0.79 ± 0.03^b^	0.82 ± 0.02^b^	0.86 ± 0.02^b^
**C20:3 (n-6)**	1.0 ± 0.0^c^	1.7 ± 0.1^b^	1.7 ± 0.0^b^	2.0 ± 0.1^a^
**C20:4 (n-6)**	17 ± 1^a^	16 ± 0^a^	14 ± 0^b^	14 ± 0^b^
**C20:5 (n-3)**	0.07 ± 0.00^a^	0.05 ± 0.00^b^	0.07 ± 0.02^ab^	0.05 ± 0.00^ab^
**C22:4 (n-6)**	0.10 ± 0.00^a^	0.10 ± 0.00^b^	0.08 ± 0.02^b^	0.10 ± 0.00^b^
**C22:5 (n-3)**	0.38 ± 0.01^b^	0.66 ± 0.01^b^	0.60 ± 0.02^b^	0.73 ± 0.03^a^
**C22:6 (n-3)**	0.39 ± 0.01^c^	0.74 ± 0.03^a^	0.54 ± 0.02^b^	0.74 ± 0.03^a^
**MUFA**	12 ± 0^b^	14 ± 0^a^	14 ± 1^a^	14 ± 0^a^
**SFA**	51 ± 1^a^	50 ± 0^a^	51 ± 1^a^	49 ± 0^b^
**PUFA**	35 ± 1^a^	32 ± 0^b^	31 ± 3^b^	32 ± 0^b^
**PUFA:SFA**	0.68 ± 0.03^a^	0.64 ± 0.01^ab^	0.60 ± 0.93^b^	0.66 ± 0.01^a^
**(n-6) PUFA**	31 ± 1^a^	27 ± 0^b^	26 ± 2^b^	27 ± 0^b^
**(n-3) PUFA**	3.6 ± 0.1^c^	4.7 ± 0.1^a^	4.2 ± 0.3^b^	5.1 ± 0.1^a^
**(n-6):(n-3) PUFA**	8.6 ± 0.1^a^	5.8 ± 0.1^c^	6.4 ± 0.32^b^	5.3 ± 0.1^c^

Data represent mean ± SEM (n = 8/group). Means within the same row that do not share a common letter are significantly different (p < 0.05). The relative proportions of fatty acids from 12:0 to 24:1(n-9) were measured, but only the major fatty acids were reported. Abbreviations used: SFA, sum of saturated fatty acids; MUFA, sum of monounsaturated fatty acids; (n-6) PUFA, sum of (n-6) polyunsaturated fatty acids; (n-3) PUFA, sum of (n-3) polyunsaturated fatty acids.

### 2.4. Splenocyte Phenotypes

Compared to lean Ctl rats, obese Ctl rats had a lower (P < 0.05) proportion of CD3^+^ (T-cells), CD3^+^CD4^+ ^(T-helper cells), CD3^+^CD8^+ ^(cytotoxic T-cells (CTLs)), CD4^+^CD28^+ ^(T-helpers cells expressing co-stimulatory molecule), CD4^+^CD45RC^-^ (mature T-helper cells), CD8^+^CD28^+ ^(activated CTL), CD8^+^CD45RC^-^ (mature CTL), OX62^+^OX6^+ ^(dendritic cell), CD45RA^+^OX6^+^ (activated B-cell) and OX6^+ ^(antigen presenting cells). Obese Ctl-fed rats also had a higher percentage of CD4^+^CD25^+^ (T-helpers cell expressing IL-2 receptor) and CD4^+^CD45RC^+^ (naïve T-helper cells) cells (Table 4, P < 0.05).

Compared to obese Ctl rats, obese EA- and VA-fed rats had a higher (P < 0.05) percentage of CD3^+^CD8^+^ and CD8^+^CD45RC^-^ (mature CTL) cells. VA-fed rats had a lower (P < 0.05) proportion of OX62^+^OX6^+ ^(dendritic cells); whereas spleens from EA-fed rats had a higher percentage of OX62^+^OX6^+ ^compared to obese Ctl rats (P < 0.05). Only obese VA-fed rats had a higher proportion of CD8^+^CD28^+ ^(CTL expressing co-stimulatory) and a lower percentage of CD4^+^CD45RC^+^ (antigen naïve T-helper cells), and CD45RA^+^OX6^+^ (activated B-cells) relative to obese Ctl rats (P < 0.05, Table 4). Obese rats fed EA had a lower proportion of OX6^+^CD11b/c^+^ (macrophages expressing MHC class II molecule) in their spleens compared to obese Ctl rats (P < 0.05, Table 4).

**Table 4:** Splenocyte phenotypes of lean Ctl rats and obese JCR:LA-*cp* rats fed VA, EA or Ctl diet.

	Lean Ctl	Obese Ctl	VA	EA
	% of gated cells			
**CD3^+^**	48 ± 1**^a^**	45 ± 1**^b^**	43 ± 1**^b^**	44 ± 1**^b^**
**CD3^+^CD4^+^**	38 ± 1**^a^**	34 ± 1**^b^**	33 ± 1**^b^**	33 ± 1**^b^**
**CD3^+^CD8^+^**	8.9 ± 0.3**^a^**	4.8 ± 1.0**^b^**	8.2 ± 0.3**^a^**	9.7 ± 0.3**^a^**
**CD4^+^CD25^+^**	5.6 ± 0.5**^b^**	7.5 ± 0.3**^a^**	8.0 ± 0.3**^a^**	4.6 ± 0.1**^c^**
**CD4^+^CD28^+^**	36 ± 1**^a^**	30 ± 1**^b^**	32 ± 1**^b^**	NM
**CD4^+^CD45RC^+^**	3.9 ± 0.57**^c^**	6.8 ± 0.42**^a^**	5.6 ± 0.25**^b^**	3.4 ± 0.20**^c^**
**CD4^+^CD45RC^-^**	40 ± 1**^a^**	37 ± 1**^b^**	36 ± 0**^b^**	35 ± 1**^b^**
**CD8^+^CD25^+^**	1.3 ± 0.1**^b^**	1.1 ± 0.1**^b^**	3.1 ± 0.1**^a^**	1.0 ± 0.0**^b^**
**CD8^+^CD28^+^**	7.6 ± 0.1**^a^**	3.5 ± 0.7**^b^**	7.6 ± 0.3**^a^**	NM
**CD8^+^CD45RC^+^**	7.0 ± 1.4**^ab^**	6.2 ± 1.0**^ab^**	7.7 ± 0.1**^a^**	4.5 ± 0.2**^b^**
**CD8^+^CD45RC^-^**	11 ± 0.4**^a^**	9.4 ± 0.3**^b^**	11 ± 0.3**^a^**	10 ± 0.4**^a^**
**CD11b/c^+^OX6^+^**	9.9 ± 0.6**^a^**	10 ± 1**^a^**	9.3 ± 0.5**^a^**	4.0 ± 0.2**^b^**
**OX62^+^OX6^+^**	1.4 ± 0.03**^b^**	1.2 ± 0.07**^c^**	1.5 ± 0.09**^b^**	1.7 ± 0.06**^a^**
**CD45RA^+^OX6^+^**	22 ± 1**^a^**	19 ± 1**^b^**	15 ± 1**^c^**	19 ± 1**^b^**
**OX6^+^**	30 ± 1**^a^**	28 ± 1**^b^**	27 ± 1**^b^**	26 ± 1**^b^**

Data represent mean ± SEM; n = 8/group. Values are a proportion of the total gated cells as determined by immunofluorescence. Differences among groups were determined by a one way ANOVA Means within the same row that do not share a common letter are significantly different (p < 0.05) as determined by Duncan’s multiple range test. NM, not measured.

### 2.5. Cytokines

**Figure 1:**
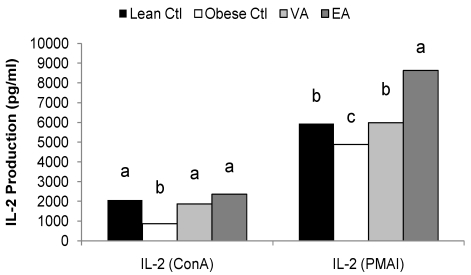
Mitogen-stimulated IL-2 production. Bars represent mean ± SEM, n=8/group. Bars not sharing a common letter are significantly different (P < 0.05). Ctl, control; VA, vaccenic acid; EA, elaidic acid.

Obese rats fed the Ctl diet produced less ConA-stimulated IL-1β, IL-2 and TNF-α and more IL-6 compared to lean rats (Figure 1 and Table 5, P < 0.05). PMA+I-stimulated IL-2 and IFN-γ was also lower in obese Ctl rats (Figure 1 and Table 5, P < 0.05). There was no significant difference in the cytokine response to LPS by splenocytes between lean and obese rats fed the control diet.

**Figure 2:**
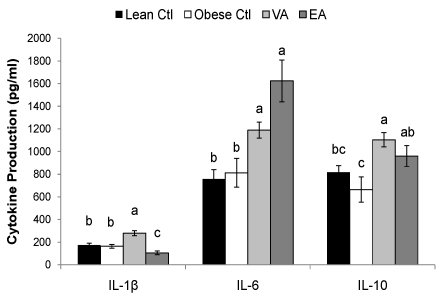
LPS-stimulated cytokine production. Bars represent mean ± SEM, n=8/group. Bars not sharing a common letter are significantly different (P < 0.05). Ctl, control; VA, vaccenic acid; EA, elaidic acid. TNF-α production did not differ among groups (458 ± 42, n = 32).

Splenoctyes of EA-fed obese animals produced more Con A and PMA+I-stimulated IL-6 as compared to VA and Ctl-fed rats (Table 5, P < 0.05). Compared to obese Ctl-fed rats, obese rats fed VA or EA produced more T-cell (ConA and PMA+I) stimulated IL-2 and TNF-α and more LPS-stimulated IL-6 and IL-10 (Figure 1 and Table 5, P < 0.05). Splenocytes of obese rats fed VA secreted more ConA-stimulated IL-4, and PMAI-stimulated IL-10 compared to obese Ctl rats, and these responses did not differ from lean Ctl rats (Table 5, P < 0.05). VA-fed rats also produced more LPS-stimulated IL-1β; whereas, EA-fed rats produced less (Figure 2, P < 0.05). 

**Table 5:** Mitogen-stimulated cytokine production of lean rats and obese JCR:LA-*cp* rats fed Ctl, VA or EA diet.

		Lean Ctl	Obese Ctl	VA	EA
		(pg/ml)			
**ConA**	**IL-1β**	73 ± 5^a^	32 ± 6.0^b^	59 ± 4.3^a^	26 ± 7.2^b^
	**IL-4**	30 ± 6.4^ab^	16 ± 3.2^b^	40 ± 4.2^a^	17 ± 2.5^b^
	**IL-6**	50 ± 7.7^c^	100 ± 15^b^	118 ± 16^b^	283 ± 47^a^
	**IL-10**	489 ± 62^a^	348± 65^a^	510 ± 40^a^	499 ± 50^a^
	**TNF-α**	130 ± 24^a^	40 ± 9.0^b^	92 ± 11^a^	83 ± 11^a^
	**IFN-γ**	670 ± 228^a^	310 ± 82^a^	416 ± 35^a^	341 ± 45^a^
**PMAI**	**IL-1β**	91 ± 7.3^a^	86 ± 9.9^a^	99 ± 10^a^	70 ± 11^a^
	**IL-6**	250 ± 22^ab^	227 ± 33^b^	218 ± 25^b^	398 ± 71^a^
	**IL-10**	2559 ± 540^ab^	1414 ± 310^b^	3599 ± 523^a^	2239 ± 391^ab^
	**TNF-α**	829 ± 107^a^	620 ± 89^a^	812 ± 76^a^	912 ± 175^a^
	**IFN-γ**	7876 ± 1002^a^	4601 ± 1032^b^	6393 ± 762^ab^	4779 ± 745^b^

Data represent mean ± SEM; n = 8/group. Means within the same row that do not share a common letter are significantly different (p < 0.05).

### 2.6. Serum Haptoglobin

Obese Ctl-fed rats had approximately three fold greater serum concentration of haptoglobin compared to lean rats (1.08 ± 0.09 mg/ml vs. 0.38 ± 0.05 mg/ml, P < 0.05). Relative to obese Ctl rats, VA-fed rats had lower serum concentrations of haptoglobin (0.52 ± 0.06 mg/ml vs. 1.08 ± 0.09 mg/ml, P < 0.05) that did not differ significantly from lean rats. Serum haptoglobin concentration of EA-fed rats did not differ from obese Ctl rats (1.30 ± 0.15mg/ml vs.1.08 ± 0.09 mg/ml, P < 0.05), but was significantly higher than lean Ctl and obese VA-fed rats. 

### 2.7. Discussion

In the present study, obese JCR:LA-*cp* rats had elevated serum haptoglobin concentrations, indicating a heightened systemic inflammatory state similar to that reported in overweight-obese patients [31]. A positive correlation between serum inflammatory markers and TFA has been reported in the literature [6,7,8]; however, the contribution of individual TFA to inflammation in obesity is not known. In the present study, obese animals fed VA had lower serum haptoglobin concentrations relative to those fed the Ctl diet and these levels did not differ from lean animals. These findings are consistent with our previous report showing a trend of VA to lower serum haptoglobin after three weeks of feeding [27]. Serum concentrations in EA-fed rats were significantly higher relative to VA-fed rats, but levels did not differ from obese Ctl rats. This suggests that EA may not independently exacerbate obesity-associated inflammation, which is reported to contribute to the development and progression of insulin resistance [31], type 2 diabetes [10], and CVD [11]. Plasma concentrations of 18:2(n-6), 20:3(n-6) and total PUFA were greater in the VA group, suggesting a possible improvement in essential fatty acid status in the VA group as compared to the EA and obese Ctl groups.

Splenocytes of obese JCR:LA-*cp* rats had an impaired ability to respond to T-cell mitogen stimulation (IL-2 and IFN-γ production), which may have been due to the lower proportion of T-helper cells (CD3^+^CD4^+^) and is consistent with previous reports [18,19,26,32,33,34,35,36]. Additionally obese rats had a higher proportion of T helper cells expressing IL-2 (CD4^+^CD25^+^) suggesting that T cells were in a state of activation in-vivo. In other chronic inflammatory disease states, T-cells that display an activated Th1 phenotype are less responsive to mitogen or antigen stimulation [37]. 

It is well established that altering that fatty acid composition of immune cell PL, particularly long chain PUFA, induces structural and functional changes in T-cells [30]. In splenocytes from obese Ctl rats, there was a lower concentration of 18:2(n-6) in both PC and PE compared to lean Ctl-fed rats. Although the concentration of arachidonic acid (AA, 20:4(n-6)) did not differ in the membrane PL there was a significantly lower concentration of AA in plasma PL, suggesting alteration in essential fatty acid status in the obese state. Additionally, total n-3 PUFA, specifically 22:5(n-3) and 22:6 (n-3), were higher in both membrane PL in obese animals compared to lean animals. Alterations in immune cell membrane concentrations of (n-6) and (n-3) PUFA have been shown to modify IL-2 production after membrane stimulation [38]. 

In the present study, feeding either TFA corrected the impaired IL-2 and TNF-α response to T-cell mitogen stimulation in obese rats. In addition, feeding VA normalized T-cell stimulated IL-1β, and IFN-γ production. This suggests that habitual consumption of the naturally occurring VA may improve impaired T-cell function in obesity. Despite the improved IL-2 and TNF-α response, EA-fed rats produced more IL-6 than the Ctl or VA-fed groups in response to T-cell mitogen stimulation. The greater production of IL-6 in response to T-cell stimulation was associated with greater incorporation of EA into splenocyte PL and indicating that EA facilitates a pro-inflammatory T-cell response. The direct impact of EA on inflammatory T-cell responses has not been examined. However, epidemiological studies and a short-term intervention study in healthy men have reported that EA and a mixture of synthetic TFA negatively impact serum inflammatory markers, including IL-6 [6,7,8]. Moreover, Han *et al* reported greater LPS-stimulated IL-6 production when overweight subjects consumed partially hydrogenated margarine [9]. These results suggest that EA augments the inflammatory response of T-cells through direct incorporation into the plasma membrane and/or through modification of intracellular targets, such as peroxisome proliferator activated receptor-gamma (PPAR-γ) [39]. In addition, we did observe that spleens of EA-fed rats weighed more than Ctl rats, but there was no significant difference in the total number of immune cells isolated. Further studies are needed to determine what caused the higher spleen weight.

In the present study, we did not directly study the mechanisms that contributed to improved T-cell stimulated cytokine responses in the VA-or EA-fed obese rodents. However, it appeared that although VA and EA were incorporated to similar levels in both PL fractions, VA and EA may act through different mechanisms. More specifically, changes induced by VA were associated with alterations in essential fatty acid composition. On the other hand, changes induced by EA may be through direct membrane incorporation and/or through intracellular targets downstream of the plasma membrane. Interestingly, the biological derivative of VA, *c*9*t*11 CLA, was not detected in either PL fraction in VA-fed rats, indicating that VA is not readily converted to CLA and/or that the CLA is not incorporated into the PLs analyzed. VA-fed rats had significantly less docosahexaenoic acid (DHA, 22:6(n-3)) and lower total (n-3) PUFA in splenocyte membrane PL compared to EA and Ctl-fed animals, and these levels were more similar to lean Ctl rats. High incorporation of long chain (n-3) fatty acids into PL membranes has been shown to reduce the inflammatory response [30]. Thus, normalization of DHA in splenocyte membranes of obese VA rats may have contributed to the improved T-cell response (as this is suppressed by a pro-inflammatory response). In contrast, the DHA or total (n-3) PUFA composition of EA-fed rat splenocytes did not differ from obese Ctl rats. Thus, the heightened T-cell response observed in EA-fed rats do not appear to be associated with changes in other long chain PUFA, but rather may be related to the direct incorporation if EA into the membrane. Moreover, EA-fed rats produced approximately 30% higher levels of IL-2 in response to PMA+I, a mitogen that bypasses the plasma membrane T-cell receptor and activates protein kinase C [40]. This suggests that EA may also target intracellular pathways downstream of the T-cell receptor.

We next examined the ability of the innate immune system to respond to LPS stimulation. Despite an impaired ability to respond to T-cell mitogen stimulation, cytokine production in response to LPS did not significantly differ with obesity in rats fed the Ctl diet. These findings are supported by our previous reports and infer that the ability of systemic innate immune cells to produce inflammatory cytokines is not impaired in obese JCR:LA-*cp* [25,18]. Feeding either TFA to obese rats resulted in greater IL-6 and IL-10 production, compared to Ctl rats after LPS-stimulation, implying that both EA and VA facilitate the production of pro-inflammatory cytokines by macrophages, dendritic cells and/or B-cells. This appears to be a long-term feeding effect as the shorter term (3 wk) dietary VA intervention did not alter LPS-stimulated IL-1β or IL-6 production in obese JCR:LA-*cp* rats [25]. In the present study, we observed differential effects of VA and EA on LPS-stimulated IL-1β production, such that cells from EA-fed rats produced less IL-1β compared to VA-fed rats. These differences may be related to our observation that EA-fed rats had fewer macrophages expressing the MHC class II molecule (OX6^+^CD11b/c^+^), which are major producers of IL-1β [41]. Long chain PUFA significantly influence the ability of inflammatory innate immune cells to respond to stimulation [30], and it is possible that the lower proportion of DHA and total (n-3) PUFA in the splenocyte PL of VA-fed rats contributed to the higher IL-1β response in contrast to EA-fed animals. However, similar changes in (n-3) PUFA composition were not observed in EA-fed rats, suggesting that other membrane or intracellular modifications may be responsible. Therefore, further investigation into the effects of these TFA may be warranted to explore these and other membrane changes in relationship to immune function in the obese state. 

## 3. Experimental Section

All animal care and experimental protocols were conducted in accordance with the Canadian Council on Animal Care and approved by the Livestock Animal Use and Care Committee at the University of Alberta. Male obese (*cp*/*cp*) and lean (+/+ or +/*cp*) rats of the JCR:LA-*cp* strain were raised in our breeding colony at the University of Alberta. Male rats only were used for this study due to the limited availability of female rats in our breeding colony. All animals were individually housed in a temperature and humidity controlled environment with a 12/12-h reversed light cycle.

Animals were weaned at 3 weeks of age and given free access to water, and standard laboratory rat diet (Lab diet 5001, PMI Nutrition International, Brentwood, MO, USA). At 8wks, male *cp*/*cp* rats were randomly allocated to receive one of the following nutritionally complete isocaloric diets (n = 8/diet) for 16 weeks: control (Ctl, 1% w/w cholesterol), VA (1% w/w VA + 1%w/w cholesterol) or EA (1%w/w EA + 1% w/w cholesterol); lean (*Cp/Cp* or *Cp/cp*) rats (n = 8) were allocated to the Ctl diet + 1% w/w cholesterol for 16 weeks. Rats were introduced to the experimental diets prior to onset of established immune abnormalities (>8 wk, unpublished data) for a 16 wk period to determine the effects of long-term exposure of TFA on immune function during the progression of obesity, insulin resistance and associated disease pathology. The non-lipid nutrient composition of the experimental diets has been published previously [19] and the fatty acid composition of the dietary lipids is provided in Table 6. Diet was prepared weekly and extruded into pellets, dried at room temperature, and stored at 4ºC in air-tight containers. Rats were housed at a room temperature of 22˚C with 60% relative humidity. Rats were weighed and feed consumption, adjusted for food spillage, was recorded twice weekly. After an overnight fast, rats were killed by cardiac exsanguination under iso-fluorane anaesthesia. Blood was collected via cardiac puncture in BD Vacuntainer^®^ (BD Biosciences, Mississauga, ON, Canada) and serum stored at -80°C. The spleen was removed under aseptic conditions.

**Table 6:** Fatty acid composition of experimental diets.

	Control	VA	EA
	% fatty acids		
**16:0**	9.1	8.9	9.2
**18:0**	47	47	35
**18:1 *trans*9**	0	0	6.7
**18:1 *trans*11**	0	6	0
**18:1 *cis*9**	17	9	16
**18:2 (n-6)**	23	25	24
**18:3 (n-3)**	1.6	1.9	1.9
**PUFA**	25	27	26
**SFA**	57	57	45
**PUFA:SFA**	0.4	0.5	0.6
**(n-6) PUFA**	23	25	24
**(n-3) PUFA**	1.6	1.9	1.9
**(n-6):(n-3) PUFA**	15	13	14

Abbreviations used: SFA, sum of saturated fatty acids; MUFA, sum of monounsaturated fatty acids; (n-6) PUFA, sum of (n-6) polyunsaturated fatty acids; (n-3) PUFA, sum of (n-3) polyunsaturated fatty acids.

### 3.1. Isolation of Splenocytes and Primary Culture Conditions

Spleens were weighed and placed in sterile 0.5% w/v bovine serum albumin in Krebs-Ringer HEPES buffer (pH 7.4) and splenocytes isolated as previously described [42]. Isolated splenocytes were re-suspended in complete culture media (RPMI 1640 supplemented with 5% (v/v) heat-inactivated fetal calf serum, 1% (v/v) antimycotic-antibiotic solution, HEPES (25 mmol/L), and 2-mercaptoethanol (2.5 mmol/L)) and cell number counted on a hemacytometer (Fisher Scientific, Edmonton, AB , Canada). Splenocytes (1.25x10^6^ cells/L) were resuspended in the culture media described above and incubated in 4 ml sterile polystyrene tubes in a humidified atmosphere at 37 °C in the presence of 5% v/v CO_2_. Splenocytes were cultured without mitogen (unstimulated cells) or with Conconavalin (ConA, 2.5mg/L), phorbal myristate acetate (PMA, 20µg/L, + Ionomycin (I, 0.5nmol/L) or LPS (1 mg/L). After 48 h of culture, the supernatant was removed and stored at -80º C for cytokine analysis. 

### 3.2. Phenotype Analysis

Immune cell subsets in freshly isolated splenocytes were identified by one, two or three colour direct immunofluorescence assays, as previously described [43]. The following pre-labelled mAbs were used: CD28, CD25, CD45RC and, OX6 (FITC-labelled); CD4 (APC-labelled); CD3, OX62 and, CD11b/c (PE-labelled); and CD8 and CD45RA (RPE-Cy-5). All antibodies were purchased from BD Biosciences, PharMingen (Mississauga, ON, Canada) except CD4, and CD45RC, which were purchased from Serotec (Cedarlane Laboratories Ltd, Hornby, ON, Canada). After a final wash, supernants were aspirated and 200 µl of cell fix (1% w/v paraformaldehyde) was added to the pellet in each well. The proportion of cells that were positive for each marker was determined by flow cytometry (FACScan; Becton Dickinson, Sunnyvale, CA) according to the relative fluorescence intensity using CellQuest software (Becton-Dickinson). Data is expressed as a proportion of splenocytes. 

### 3.3. Cytokine Production and Serum Haptoglobin

The cultured cell supernatants of unstimulated, ConA, PMA+I and LPS-stimulated splenocytes were used to determine the concentration of TNF-α (detection limit: 31.2–2000pg/ml), IL-4 (1.6#x2013;100 pg/mL), IL-6 (78#x2013;5000 pg/mL), IL-10 (15.6#x2013;1000 pg/mL) and IFN-γ (31.25#x2013;2000 pg/mL) at concentrations as per manufacturers instructions for OptEIA anti-rat enzyme-linked immunoasorbant assays (ELISA) (BD Biosciences, PharMingen, Mississauga, ON, Canada), and with DuoSet ELISA for IL-1β (31.2#x2013;2000 pg/mL) and IL-2 (23.4#x2013;1500 pg/mL) (R&D Systems, Cedarlane, Missassauga, ON, Canada). Values below the range of detection were assigned half of the value of the lowest standard value. Serum haptoglobin levels were determined by a colorimetric assay (Tri-Delta Development Ltd, Maynooth, Ireland). All samples were measured in duplicate and the absorbance for individual cytokines and haptoglobin were measured at 450 nm and 630 nm, respectively, on a microtitre plate reader (SPECTRAmax 190, Molecular Devices, Sunnyvale, CA). The coefficient of variance was ≤ 10% for all assays and the mean of duplicates was used for statistical analysis.

### 3.4. Plasma and Splenocyte PL Fatty Acid Composition

Lipids were extracted from plasma and splenocytes by a modified Folch method as previously described [44]. Total PL were separated on silica G plates as previously described [45] and individual PL were separated on thin layer chromatography plates (HPK silica gel 60nm 10 × 10cm; Fisher Scientific, Edmonton, AB, Canada), and visualized with 8-anilino-1-naphthalenesulfonic acid under UV light against the appropriate standards. Fatty acid methyl esters were prepared from the scraped silica bands of total plasma PL and splenocyte phosphatidylcholine (PC) and phosphatidylethanolamine (PE) [44] and separated by automated gas liquid chromatography (Varian 3800, Varian Instruments, Mississauga, ON, Canada) using a 100m *CP*-Sil 88 fused capillary column (Varian Instruments, Mississauga, ON, Canada) as described previously [46].

### 3.5. Statistics

Statistical analysis was conducted using one-way ANOVA on the SAS software statistical package (Version 9.1, SAS Institute, Cary, NC). All data was reported as mean ± standard error of the mean (SEM). Significant differences among groups were determined by Duncan’s multiple range test (P < 0.05) and all non-parametric data was log-transformed prior to statistical analyses.

## 4. Conclusions

In conclusion, obese JCR:LA-*cp* rodents display similar immune abnormalities reported in obese humans. We observed higher serum concentrations of haptoglobin, which may have contributed to the suppression of T cell proliferation (IL-2 production) in cells from obese animals. Feeding either TFA improved many T-cell stimulated cytokine concentrations. However, only VA normalized serum haptoglobin and this may have contributed to improved T-cell stimulated cytokine responses. Incorporation of VA and improvements in essential fatty acid composition in membrane and plasma PL may improve systemic inflammation and T-cell responses in VA-fed rats. Splenocytes of EA-fed rats produced more IL-6 in response to a T-cell mitogen, suggesting that EA facilitates inflammation and may impair the ability of T-cells to regulate the inflammatory response. Feeding either EA or VA induced greater IL-6 and IL-10 production, indicating that both the naturally and synthetically derived TFA can facilitate LPS-stimulated immune responses. Our data suggest that VA may induce cytokine changes by modifying membrane PL essential fatty acid composition; whereas, EA may act through direct incorporation into membrane PL and/or intracellular targets. Further research is required to understand the implications of these findings as they relate to TFA, PL incorporation and inflammatory immune responses in the modulation of the pro-inflammatory state in obesity, insulin resistance and type 2 diabetes. 
